# Supplementary material for: STGAD: Self-temporal generative adversarial framework with transformer attention for unsupervised multivariate time-series anomaly detection and localization
Source: PLoS One. 2026 May 21;21(5):e0349223. doi: 10.1371/journal.pone.0349223 (PMC13193399; doi:10.1371/journal.pone.0349223)
Supplement: Attachment — Submitted filename: Response to Reviewers.docx [file pone.0349223.s002.docx]

**Revised Response Letter:**

**Dear Editor,**

**Response to Reviewers (Reviewer #1 & Reviewer #2)**

We sincerely thank both reviewers for their time and constructive feedback, which has greatly contributed to improving the quality of our manuscript. In this revision, we have made significant changes in response to the concerns raised, focusing on improving clarity, consistency, and addressing specific points outlined in their feedback.

Below are the detailed point-by-point responses:

**Reviewer #1**

**Major Concerns:**

1. **Manuscript Length and Readability:**
   - **Response:** We have streamlined the manuscript by removing repetitive explanations on datasets, windowing, and model design. We also simplified the language, improved grammatical accuracy, and standardized the notation to enhance readability.
2. **Generator and Non-conditional Architecture:**
   - **Response:** We clarified that the generator in STGAD is non-conditional and generates sequences from latent noise without conditioning on the input. During inference, we sample multiple generated sequences and select the one with the minimum residual to reduce variability, providing a more stable reconstruction.
3. **Window Length:**
   - **Response:** We included a sensitivity study for window length L. Our results show that smaller window lengths (L ≤ 20) lead to strong performance, while very large window lengths (L > 50) degrade performance due to diluting localized anomaly signals. We justify using L = 5 based on these findings.
4. **Baselines’ Preprocessing and Hyperparameters:**
   - **Response:** We provided further details on the baseline models’ training protocol, confirming that they underwent identical preprocessing (z-score normalization), the same window size, and the same thresholding method (POT). We also clarify that hyperparameters for the baselines were not tuned to each dataset to ensure a fair unsupervised comparison.
5. **Computational Efficiency:**
   - **Response:** We included additional metrics on computational performance, such as training time per epoch, inference throughput, GPU memory usage, and complexity scaling with the number of variables for both STGAD and the baselines. This provides a comprehensive assessment of the model's real-world feasibility.
6. **Interpretability Section Length:**
   - **Response:** We condensed the interpretability section, focusing on key insights. We retain the SMAP case study for intuition but removed case-specific details to streamline the presentation. We also added distribution-level statistics to report overlaps for different quantile-sampled true-positive windows.

**Minor Issues:**

- **(a) Equation Motivation:**
  - **Response:** We clarified the equation terms and provided brief explanations for the generator loss, including its relation to the discriminator’s score.
- **(b) Inconsistent β values:**
  - **Response:** We fixed β to a single default (β = 0.5) throughout the experiments and provided sensitivity analysis to justify the choice.
- **(c) Low-resolution Figures:**
  - **Response:** We improved the quality of the figures and corrected formatting errors.

**Reviewer #2**

**Major Concerns:**

1. **Fusion Weight β Justification:**
   - **Response:** We revised the manuscript to clarify that β is fixed globally at β = 0.5 for all experiments. The sensitivity of β is reported to show its stability and performance robustness, without relying on dataset-specific tuning.
2. **Non-conditional Generator and Reconstruction:**
   - **Response:** We addressed the contradiction regarding the non-conditional generator by explaining the latent-to-sequence mapping and how the reconstruction error is computed by selecting the sample with the minimal residual. We explicitly acknowledged that STGAD is a hybrid method combining adversarial training with residual scoring.
3. **Self-Temporal Transformer Clarification:**
   - **Response:** We revised the description of the Self-Temporal Transformer to clarify that it is a standard Transformer encoder used in the critic to model global temporal dependencies. The term “Self-Temporal” is intended to describe its role in within-window temporal modeling, not a fundamentally new architecture.
4. **Ablation Study and Transformer’s Role:**
   - **Response:** We revised the manuscript to moderate the claims regarding the Self-Temporal Transformer, acknowledging that WGAN-GP plays a crucial role in stable adversarial training. We now emphasize the combined effectiveness of the WGAN-GP loss, temporal modeling in the critic, and dual-score fusion, which together enable STGAD’s performance.
5. **MIT-BIH Naming Convention:**
   - **Response:** We have corrected the name to "MIT-BIH" throughout the manuscript to avoid confusion with "MBA."
6. **Formatting Issues:**
   - **Response:** We have performed a thorough proofreading pass to correct formatting errors, such as line breaks, spacing, and alignment issues.
7. **Saliency Overlap Statistics:**
   - **Response:** We addressed the concern regarding Saliency-Local and Saliency-Global overlaps by adding distribution-level statistics. We report the median and interquartile range for overlaps based on quantile-sampled true-positive windows, moving away from relying on a single example.
8. **WGAN-GP and Stability Evidence:**
   - **Response:** We have included additional stability metrics to support the claim that WGAN-GP improves stability. This includes training curves, loss variance for both discriminator and generator, and quantitative diagnostics for mode collapse.
9. **Noise Level Fairness in Comparison:**
   - **Response:** We have aligned the noise levels used for STGAD and baselines, ensuring a fair comparison across all methods at identical noise intensities (σ=0.1 and σ=0.25).

We hope that these revisions have addressed the reviewers' concerns, and we look forward to your feedback. Thank you again for your valuable input.

**Kind regards,**

Faith Yihan Mu
