# Supplementary material for: STGAD: Self-temporal generative adversarial framework with transformer attention for unsupervised multivariate time-series anomaly detection and localization
Source: PLoS One. 2026 May 21;21(5):e0349223. doi: 10.1371/journal.pone.0349223 (PMC13193399; doi:10.1371/journal.pone.0349223)
Supplement: Attachment — Submitted filename: Response_to_Reviewers_auresp_2.docx [file pone.0349223.s003.docx]

**Response to Reviewers**

Dear Editor and Reviewers,

We sincerely thank you and the reviewers for your careful evaluation of our manuscript and for the constructive comments and suggestions. We have carefully considered all the points raised and revised the manuscript accordingly. In this round of revision, the main improvements include: further clarifying the novelty and design rationale of the proposed method, supplementing the discussion of efficiency and practical deployability, strengthening the justification for the window length selection, enhancing the interpretability analysis for anomaly localization, and polishing the language and figure presentation throughout the manuscript.

We believe these revisions have significantly improved the clarity, rigor, and overall quality of the manuscript. Our detailed responses to each comment are provided below.

**Reviewer #1**

We sincerely appreciate the reviewer’s positive assessment of our work and the constructive suggestions for further improvement. We are encouraged that the reviewer recognized the importance of the problem, the overall soundness of the technical framework, and the experimental effectiveness of the proposed method on multiple benchmark datasets. In response to the reviewer’s comments, we have revised the manuscript accordingly. Our point-by-point responses are as follows.

**Comment 1:**

**Model novelty should be clarified further. The Self-Temporal Transformer appears to be a standard Transformer encoder integrated into the discriminator, so the novelty compared with existing transformer-based anomaly detection models should be more clearly emphasized.**

**Response:**

Thank you for this valuable comment. We agree that, in the previous version, the description of the “Self-Temporal Transformer” may have given the impression that it was a newly proposed Transformer architecture, which was not our intention. In the revised manuscript, we have clarified that the Self-Temporal Transformer in STGAD is based on a standard Transformer encoder embedded in the discriminator (critic), where it is used to capture temporal dependencies within each window and interactions across variables. We have also revised the relevant description to make it clearer that the contribution of STGAD lies in the overall framework design for unsupervised multivariate time-series anomaly detection, rather than in proposing a new attention module itself.

Location in revised manuscript: Abstract (p. 1); the final paragraph of the Introduction (p. 4); Section 3.2, especially “Model Architecture” and “Discriminator Architecture” (pp. 8–9); and Section 3.3, “Training and Inference” (pp. 9–10).

**Comment 2:**

**Generator design justification is limited. The use of a non-conditional generator with sample matching during inference may introduce additional computational overhead. The authors should discuss the efficiency and scalability of this approach in practical deployments.**

**Response:**

Thank you for this important suggestion. In the revised manuscript, we have expanded the discussion of the rationale behind the non-conditional generator, as well as the efficiency and scalability of the proposed inference strategy.

First, we have clarified the role of the generator in STGAD. Unlike conditional reconstruction-based methods, the generator in our framework is non-conditional and learns the distribution of normal patterns from the latent space. During inference, multiple candidate sequences are sampled for each input window, and the one with the minimum residual is selected to construct the residual-based anomaly cue. This sample-matching strategy helps mitigate the variability introduced by stochastic generation and improves the stability of the residual score.

To address the reviewer’s concern regarding computational overhead, we have added a dedicated efficiency analysis in the revised manuscript, including training time and inference-related metrics such as throughput, per-window latency, and memory usage. The results show that STGAD maintains competitive training efficiency and achieves practical inference performance, making it suitable for offline analysis and near-real-time monitoring scenarios. We further explain that the inference cost scales approximately linearly with the number of samples N, so in practical deployments, a moderate choice of N can provide a good trade-off between detection performance and computational efficiency.

In addition, we have added a discussion noting that the current design is particularly suitable for industrial monitoring and diagnostic settings where robust detection is prioritized and a moderate sampling cost is acceptable. For more stringent real-time scenarios, future work may explore reducing the number of samples or applying model compression and lightweight deployment strategies to further improve inference efficiency.

Location in revised manuscript: Abstract (p. 1); Section 3.2, especially “Model Architecture” and “Generator Architecture” (pp. 8–9); Section 3.3, “Training and Inference” (pp. 9–10); Section 4.2, “Windowing and Model Configuration” (p. 13); Section 5.1 and Tables 3–4 (pp. 15–16); and Section 5.4 with Fig. 6 (pp. 19–20).

**Comment 3:**

**Window size selection (L=5) appears relatively small. More justification or broader sensitivity analysis would strengthen the methodological choices.**

**Response:**

Thank you for this helpful suggestion. In response, we have added a sensitivity analysis on the window length L in the revised manuscript and further explained why L=5 was selected as the default setting.

In the new sensitivity experiments, we systematically examined the effect of window length on detection performance, together with other important hyperparameters such as the fusion weight β and the number of samples N. The results indicate that STGAD generally achieves stable and favorable performance under short to moderate window lengths. In contrast, excessively long windows, while containing more contextual information, may dilute localized anomaly patterns, introduce greater temporal variability, and increase computational complexity, which can negatively affect both detection effectiveness and efficiency.

Based on these observations, we have clarified in the manuscript that the choice of L = 5 was guided by both empirical evidence and practical trade-offs among detection accuracy, stability, and computational cost. The newly added sensitivity analysis provides stronger empirical support for this design choice.

Location in revised manuscript: Section 4.2, “Windowing and Model Configuration” (pp. 12–13); and Section 5.4, “Sensitivity Analysis,” together with Fig. 6 (pp. 19–20).

**Comment 4:**

**Interpretability remains limited. Although the authors include a short interpretability discussion, further insights into how anomalies are localized across variables would improve the practical usefulness of the method.**

**Response:**

Thank you for this valuable comment. We agree that interpretability is particularly important for multivariate time-series anomaly detection, especially in practical applications where practitioners are concerned not only with whether an anomaly occurs, but also with which variables contribute most to it.

To address this concern, we have further expanded the interpretability analysis in the revised manuscript. Specifically, we retained the case study based on the SMAP dataset to visually illustrate how anomalous windows can be localized across time steps and variables. More importantly, we extended the analysis beyond a single qualitative example by adding a distribution-level quantitative evaluation.

In particular, we compared the top-5 salient variables identified by the model with two model-agnostic statistical anomaly cues, namely local change magnitude and global deviation, using overlap@5 as the evaluation metric. For true positive samples under different confidence intervals, we report the median and interquartile range of the overlap values, thereby providing a more systematic assessment of the consistency between the model’s variable-level attribution and independent statistical evidence.

Through these additions, we aim to demonstrate more clearly that STGAD can provide not only anomaly scores but also meaningful variable-level localization results that are broadly consistent with independent statistical evidence, thereby improving its practical usefulness in monitoring and diagnostic tasks.

Location in revised manuscript: Section 5.5, “Interpretability Analysis” (pp. 20–22), including Figs. 7–8 (p. 21) and Tables 8–9 (p. 22).

**Comment 5:**

**Minor language and formatting issues remain and should be carefully proofread before publication.**

**Response:**

Thank you for the reminder. We have carefully proofread the entire manuscript and further improved the language and formatting in the revised version.

(1) We unified terminology and notation throughout the manuscript.

(2) We revised several sentences and paragraphs to improve clarity, readability, and precision of expression.

(3) We corrected minor issues related to formatting, spacing, line breaks, and the presentation of tables and figure captions.

(4) We improved the visual quality of the figures by replacing them with higher-quality vector formats to ensure better readability when enlarged.

We hope that these revisions have substantially improved the language quality and overall presentation of the manuscript.

Location in revised manuscript: These language and formatting revisions were applied throughout the revised manuscript (pp. 1–25), including all sections, tables, and figures.

**Reviewer #2**

We sincerely thank the reviewer for recognizing the improvements made in the previous revision. We are pleased that the reviewer considers the major concerns raised earlier to have been addressed. In this round, we have also carefully followed the reviewer’s additional suggestions and revised the manuscript accordingly.

**Comment 1:**

**The author's response has addressed my previous concerns. I recommend further polishing the English writing before formal publication.**

**Response:**

Thank you very much for your positive feedback and helpful suggestion. Following your recommendation, we have further polished the English throughout the manuscript. In particular, we improved the clarity of several long and complex sentences, refined wording in some sections to make the presentation more natural and precise, and checked the consistency of terminology and grammar throughout the paper. We believe that the revised manuscript is now clearer and more readable.

Location in revised manuscript: Further language polishing was applied throughout the revised manuscript (pp. 1–25).

**Comment 2:**

**Additionally, the resolution of the figures is currently low, which affects the reading experience; I suggest replacing all figures with vector formats.**

**Response:**

Thank you for this helpful suggestion. In response, we carefully rechecked all figures in the manuscript and replaced them with higher-quality vector formats to improve visual clarity and readability, especially when enlarged. We also re-examined the figure captions, axis labels, and layout to ensure consistency and improve the overall presentation quality of the manuscript.

Location in revised manuscript: The updated vector figures are provided in Figs. 1–8 (pp. 8–9, 13, 17, 19, and 21).

Once again, we sincerely thank the Editor and both Reviewers for their careful reading of our manuscript and for their constructive and insightful comments. In response, we have made substantial revisions to further clarify the methodological contributions, supplement the efficiency and sensitivity analyses, strengthen the interpretability discussion, and improve the language and figure quality throughout the manuscript. We hope that the revised manuscript and the present responses adequately address all concerns and that the paper is now suitable for publication.

Sincerely,

The Authors
