# Supplementary material for: STGAD: Self-temporal generative adversarial framework with transformer attention for unsupervised multivariate time-series anomaly detection and localization
Source: PLoS One. 2026 May 21;21(5):e0349223. doi: 10.1371/journal.pone.0349223 (PMC13193399; doi:10.1371/journal.pone.0349223)
Supplement: Attachment — Submitted filename: Response_to_Reviewers_auresp_3.docx [file pone.0349223.s004.docx]

**Response to Reviewers**

Dear Editor and Reviewers,

We sincerely thank you and the reviewers for your careful evaluation of our manuscript and for the constructive comments and suggestions. We have carefully considered all the points raised and revised the manuscript accordingly. In this round of revision, the main improvements include: further elaborating the practical use of the proposed anomaly localization results, clarifying the trade-off between the sampling size and inference cost in different deployment scenarios, adding a paragraph at the end of the Introduction to describe the structure of the paper, incorporating the reviewer-suggested reference at a suitable location in the Introduction, further completing and standardizing the reference list by adding DOI information where available, revising the Data Availability Statement to more accurately reflect the accessibility of each dataset, and polishing the language and formatting throughout the manuscript.

We believe these revisions have further improved the clarity, completeness, and presentation quality of the manuscript. Our detailed responses to each comment are provided below.

**Reviewer #1**

We sincerely appreciate the reviewer’s positive assessment of our revised manuscript and the constructive suggestions for further improvement. We are encouraged that the reviewer recognized the soundness of the technical framework, the comprehensiveness of the experimental evaluation, and the practical relevance of the proposed method. In response to the reviewer’s comments, we have further revised the manuscript accordingly. Our point-by-point responses are as follows.

**Comment 1:**

While interpretability has been improved, the explanation of how practitioners can directly use the localization results in real-world applications could be further elaborated.

**Response:**

Thank you for this valuable suggestion. We agree that, beyond showing whether the model can localize anomalies across variables, it is also important to clarify how such localization results can be used in real monitoring and diagnostic workflows.

In the revised manuscript, we have therefore expanded the discussion in the interpretability section to explain the practical role of variable-level localization more explicitly. In particular, we now clarify that the localization results should be interpreted as diagnostic prioritization cues rather than as fully automatic root-cause decisions. We further explain that, once an anomalous window is detected, practitioners can inspect the top-ranked variables and relate them to known sensor-to-subsystem mappings, control loops, or equipment modules. In this way, the localization output can help narrow the troubleshooting scope, prioritize targeted inspection or sensor validation, and support alarm triage in practical monitoring settings.

Through this revision, we aim to make clearer that the localization output of STGAD is intended as a decision-support signal for downstream diagnosis, thereby strengthening its practical usefulness in real-world applications.

**Location in revised manuscript:** Section 5.5, “Interpretability Analysis”; and Section 6, “Conclusion”.

**Comment 2:**

The discussion on the trade-off between sampling size (N) and inference cost could be made more explicit, especially for strict real-time deployment scenarios.

**Response:**

Thank you for this helpful suggestion. We agree that the practical implications of the sampling budget should be described more explicitly, especially for different deployment settings.

In response, we have strengthened the discussion of the trade-off between the number of generator samples N and inference cost in two places. First, in the comparative evaluation section, we now clarify that the inference latency and throughput reported in Table 4 are measured under the default sampling setting used in our experiments. We also explicitly state that, because the residual branch relies on sample matching over multiple generated candidates, the inference cost increases approximately linearly with the sampling budget N. Second, in the sensitivity analysis section, we further explain that N serves as a controllable knob for balancing residual robustness against inference cost under different operational requirements.

We now distinguish more clearly among offline analysis, near-real-time monitoring, and stricter real-time scenarios. Specifically, we explain that a moderate N is preferable in offline or post-event analysis because it provides a more stable residual estimate, whereas in stricter real-time deployments, N can be reduced to satisfy tighter latency constraints, with the understanding that this may slightly weaken the robustness of the sample-matching residual.

These additions make the deployment-oriented interpretation of the sampling budget more explicit and directly address the reviewer’s concern.

**Location in revised manuscript:** Section 5.1, “Comparative Evaluation with Baseline Methods,” especially the discussion following Table 4; Section 5.4, “Sensitivity Analysis”; and Section 6, “Conclusion”.

**Comment 3:**

Some minor language and grammatical issues may still remain and could benefit from one final round of careful proofreading.

**Response:**

Thank you for the reminder. We have carefully proofread the manuscript again and further polished the language and formatting throughout the revised version.

In particular:

(1) We corrected minor grammatical and stylistic issues in multiple sections.

(2) We unified terminology and presentation across the manuscript, including section text, tables, and figure captions.

(3) We corrected minor formatting inconsistencies, including punctuation, spacing, abbreviation usage, and table/figure title formatting.

(4) We further checked the consistency of the revised reference list and related cross-references in the main text.

We hope that these revisions have further improved the readability and overall presentation quality of the manuscript.

**Location in revised manuscript:** These language and formatting revisions were applied throughout the revised manuscript.

**Reviewer #3**

We sincerely thank the reviewer for the helpful suggestions and for recommending specific improvements that could further enhance the presentation quality of the manuscript. In response, we have revised the manuscript accordingly. Our point-by-point responses are as follows.

**Comment 1:**

It is suggested to briefly discuss the structure of subsequent sections in a single paragraph at the end of introduction section.

**Response:**

Thank you for this helpful suggestion. We agree that a brief roadmap paragraph at the end of the Introduction can improve the readability and organization of the manuscript.

In response, we have added a short paragraph at the end of the Introduction to summarize the structure of the remainder of the paper. This newly added paragraph briefly describes the content of Sections 2–6, including the related work review, the proposed methodology, the experimental setup, the results and analysis, and the conclusion.

**Location in revised manuscript:** End of Section 1, “Introduction”.

**Comment 2:**

It is suggested to include the DOI information for each research paper in the reference section.

**Response:**

Thank you for this valuable suggestion. In response, we carefully rechecked the reference list and added DOI information for the cited research papers where DOI records are available. We also corrected and standardized several reference entries, including formatting details in conference proceedings and arXiv citations, to improve completeness and consistency.

We hope that these revisions have improved the quality and traceability of the reference list.

**Location in revised manuscript:** Reference list.

**Additional suggestion:**

For suggestion 1 to 2, author can refer and cite the paper titled “Multilingual entity alignment by abductive knowledge reasoning on multiple knowledge graphs” at a suitable location.

**Response:**

Thank you for this helpful recommendation. We reviewed the suggested paper carefully and agree that it can be cited as an example of recent engineering AI work emphasizing structured relational reasoning on complex heterogeneous data.

Accordingly, we have incorporated this reference at a suitable location in the Introduction. Specifically, we added it to the discussion of recent attention-based, graph-based, and hybrid modeling trends, where it is cited to support the broader point that structured relational reasoning is increasingly important in advanced engineering AI applications.

**Location in revised manuscript:** Section 1, “Introduction,” in the paragraph discussing attention-based, graph-based, and hybrid modeling approaches.

**Additional clarification on data availability:**

In addition to the comments above, we also re-examined the Data Availability Statement to ensure that it accurately reflects the accessibility of each dataset used in this study. In the revised manuscript, we now distinguish more clearly between datasets that are publicly accessible and the SWaT dataset, which is available upon request from its provider. We believe this revision provides a more accurate and transparent description of the data sources used in the study.

**Location in revised manuscript:** Data Availability Statement.

Once again, we sincerely thank the Editor and the Reviewers for their careful reading of our manuscript and for their constructive and insightful comments. In response, we have made further revisions to improve the practical interpretation of anomaly localization, clarify deployment-related trade-offs, strengthen the organization of the Introduction, improve the completeness of the reference list, refine the Data Availability Statement, and further polish the language and formatting throughout the manuscript. We hope that the revised manuscript and the present responses adequately address all concerns and that the paper is now suitable for publication.

Sincerely,

The Authors
